# Time-Course Changes of Oxidative Stress Response to High-Intensity Discontinuous Training versus Moderate-Intensity Continuous Training in Masters Runners

**DOI:** 10.1371/journal.pone.0087506

**Published:** 2014-01-31

**Authors:** Alessandra Vezzoli, Lorenzo Pugliese, Mauro Marzorati, Fabio Rubens Serpiello, Antonio La Torre, Simone Porcelli

**Affiliations:** 1 Institute of Bioimaging and Molecular Physiology, CNR, Segrate (Milano), Italy; 2 Department of Biomedical Sciences for Health, Università degli Studi di Milano, Milano, Italy; 3 Institute of Sport, Exercise and Active Living (ISEAL), College of Sport and Exercise Science, Victoria University, Melbourne, Australia; 4 Department of Medical and Biological Sciences, Università degli Studi di Udine, Udine, Italy; University of Arkansas for Medical Sciences; College of Pharmacy, United States of America

## Abstract

Beneficial systemic effects of regular physical exercise have been demonstrated to reduce risks of a number of age-related disorders. Antioxidant capacity adaptations are amongst these fundamental changes in response to exercise training. However, it has been claimed that acute physical exercise performed at high intensity (>60% of maximal oxygen uptake) may result in oxidative stress, due to reactive oxygen species being generated excessively by enhanced oxygen consumption. The aim of this study was to evaluate the effect of high-intensity discontinuous training (HIDT), characterized by repeated variations of intensity and changes of redox potential, on oxidative damage. Twenty long-distance masters runners (age 47.8±7.8 yr) on the basis of the individual values of gas exchange threshold were assigned to a different 8-weeks training program: continuous moderate-intensity training (MOD, n = 10) or HIDT (n = 10). In both groups before (PRE) and after (POST) training we examined the following oxidative damage markers: thiobarbituric acid reactive substances (TBARS) as marker of lipid peroxidation; protein carbonyls (PC) as marker of protein oxidation; 8-hydroxy-2-deoxy-guanosine (8-OH-dG) as a biomarker of DNA base modifications; and total antioxidant capacity (TAC) as indicator of the overall antioxidant system. Training induced a significant (p<0.05) decrease in resting plasma TBARS concentration in both MOD (7.53±0.30 and 6.46±0.27 µM, PRE and POST respectively) and HIDT (7.21±0.32 and 5.85±0.46 µM, PRE and POST respectively). Resting urinary 8-OH-dG levels were significantly decreased in both MOD (5.50±0.66 and 4.16±0.40 ng mg^−1^creatinine, PRE and POST respectively) and HIDT (4.52±0.50 and 3.18±0.34 ng mg^−1^creatinine, PRE and POST respectively). Training both in MOD and HIDT did not significantly modify plasma levels of PC. Resting plasma TAC was reduced in MOD while no significant changes were observed in HIDT. In conclusion, these results suggest that in masters runners high-intensity discontinuous does not cause higher level of exercise-induced oxidative stress than continuous moderate-intensity training, inducing similar beneficial effects on redox homeostasis.

## Introduction

During exercise, the high energy demand required by muscle contraction causes an increase of oxygen (O_2_) delivery/uptake, leading to an increase of O_2_ consumption up to 200-fold compared to rest in the muscle fibres [1]. The high O_2_ flux along the mitochondrial electron transport chain, in association with an electron leakage [2]–[4], is correlated with an increased production of free radicals and reactive oxygen and nitrogen species (ROS) [2], [5], [6]. This phenomenon, usually defined as exercise-induced oxidative stress, has been implicated in the damage of cellular membranes, increased cellular swelling, decreased cell membrane fluidity, and DNA damage [7]–[9]. In skeletal muscle fibres, exercise-induced oxidative stress is also linked to fatigue, longer recovery time and increased injury rate [10]–[12]. Indeed ROS can modify sarcoplasmic reticulum calcium handling, acting on calcium-release channels and SERCA, and alter structure and function of myofilaments [13].

It has been demonstrated that exercise intensity plays an important role in ROS production by modulating the level of exercise-induced oxidative stress [14], [15]. During aerobic exercise, the generation of ROS increases according to a higher O_2_ consumption and, consequently, a higher electron leakage from the electron transport chain [16]. If ROS generation exceeds antioxidant defenses (i.e. when exercise intensity is greater than 60–70% of maximal oxygen uptake) oxidative damage is observed [17].

Nevertheless, the association between exercise and oxidative stress is not always negative. The chronic repetition of exercise, i.e. exercise training, may have the capability to develop a compensation to oxidative stress in skeletal muscle fibres [18] by means of an adaptation of the antioxidant and repair systems. This might result in a decreased resting level of oxidative damage and an increased resistance to oxidative stress [19]–[22]. Several studies have demonstrated that antioxidant enzymes adaptation is one of the fundamental changes in response to exercise training within the skeletal muscle (for a review see [18]), as described for mitochondrial oxidative enzymes [23]. Indeed, increased levels of ROS and oxidative damage are initiators of specific adaptive responses, such as the activation of antioxidant enzymes [24] and enhanced oxidative damage repair [20]. The effects of training on oxidative stress depend on training characteristics (i.e., intensity, type, volume, duration) [25], [26], [27]. Several studies have demonstrated that in humans continuous aerobic training, characterized by a constant sub-maximal intensity, reduces ROS production and increases antioxidant defences [18], [28], [29]. Recently, focus has shifted toward training modalities different from the traditional continuous aerobic training, such as high-intensity discontinuous training (HIDT). This training method is characterized by brief intermittent bouts of vigorous activity interspersed by periods of rest or low intensity exercise [30]. HIDT causes repeated O_2_ consumption fluctuations related to changes of exercise intensity as opposed to continuous endurance training where O_2_ consumption is nearly constant during the exercise.

HIDT, traditionally used by athletes, it is now increasingly employed in young healthy sedentary individuals as an effective time-efficient alternative to moderate intensity continuous endurance training, inducing similar or even superior changes in a range of physiological parameters, performance and health-related markers [30]. Indeed, the benefits of HIDT extend to health promotion and are currently proposed for improving health and reducing fatigue also in middle-aged subjects and in many diseases (COPD and cardiac patients) [31]

Aging is associated with increased free radical generation in the skeletal muscle that can cause oxidative modification of protein, lipid, and DNA [32]. Research evidence indicates that senescent organisms are more susceptible to oxidative stress during exercise because of the age-related ultrastructural and biochemical changes that facilitate formation of reactive oxygen species (ROS) [33]. Aging also increases the incidence of muscle injury, and the inflammatory response can subject senescent muscle to further oxidative stress [2]. Furthermore, muscle repair and regeneration capacity is reduced at old age that could potentially enhance the accrual of cellular oxidative damage [34]. Nevertheless, the elderly who are physically active benefit from exercise-induced adaptation in cellular antioxidant defense systems [35]. Improved muscle mechanics, strength, and endurance make them less vulnerable to acute injury and chronic inflammation. Indeed, moderate levels of oxidative stress are essential for the organisms to adapt and reach a new level of hormesis even if the balance of oxidants and antioxidants becomes more fragile in advance age [36].

Up to date no study has investigated the effects of prolonged (>1 week) high-intensity discontinuous training on ROS production and exercise-induced oxidative stress in middle-age subjects. These data could be particularly relevant to older subject since it has been reported that both resting and exercise-induced free radical-mediated lipid peroxidation is more pronounced in senescent compared with young human skeletal muscle [37].

The aim of this study was to evaluate the effects of 8-week high-intensity discontinuous training (HIDT) on resting level and time-course changes of several indexes of oxidative stress in masters runners. Since HIDT is characterized by repeated variations of intensity associated with changes of redox potential, ATP/ADP ratio and, consequently, disturbances of cellular homeostasis [38], we hypothesised that HIDT might cause a higher level of exercise-induced oxidative stress compared to a workload-matched, moderate-intensity continuous training (MOD).

## Methods

### Participants

Twenty healthy masters runners volunteered to participate in this study. The physical and physiological characteristics of the participants are shown in Table 1. They were all male athletes, competing at national level, with several years (21±4 years) of training experience and training habits of about 45 km wk^−1^. Participants were matched on PRE gas exchange (GET) value (see above for further details) before being stratified into two groups completing 8 weeks (3 times non consecutively per week) of moderate-intensity continuous (MOD, n = 10) or high-intensity discontinuous training (HIDT, n = 10) (see training intervention for further details). All participants signed a written consent after being informed of all risks, discomforts and benefits associated with the study. All tests were conducted in the laboratories of the Institute of Bioimaging and Molecular Physiology of the National Research Council under close medical supervision and subjects were continuously monitored by 12-lead electrocardiography (ECG). Procedures were in accordance with the Declaration of Helsinki, and institutional review board (Comitato Etico Indipendente ASL Milano Due) approval was received for this study.

**Table 1:** Physical and physiological characteristics of the participants.

	MOD	HIDT
	(n = 10)	(n = 10)
Age (years)	50.6±6.3	45.1±8.5
Body mass (kg)	69.6±10.1	72.2±9.1
Height (m)	1.74±0.07	1.76±0.06
BSA	1.82±0.16	1.86±0.14
Body mass index (kg m^−2^)	22.8±1.95	23.1±2.3
VO_2__peak_ (l min^−1^)	3.25±0.33	3.50±0.39
GET	2.87±0.23	3.04±0.31

BSA, body surface area; VO_2peak_, maximal oxygen consumption; GET, Gas exchange threshold.

### Experimental design

Participants underwent medical examination and were carefully instructed about the experimental procedures in a preliminary session. In the same occasion, anthropometric measures were collected and familiarisation with the testing procedures and equipment was requested. After that, subjects visited the laboratory twice (DAY1 and DAY2) both before (PRE) and at the end (POST) of training. In DAY1, participants performed an incremental test up to voluntary exhaustion (IE). In DAY2, at least 48 hours after, participants underwent two constant-load submaximal exercises (CLE). Blood and urine samples were collected: at rest (REST) in DAY1 and DAY2; and, in DAY2, immediately at the end (END), after 1 (1H) and 2 (2H) hours of CLE. Blood samples at rest were also collected after 4 weeks (4WK). During all the experimental period was recommended to keep unchanged dietary habits, in particular oxidant and antioxidant food (diet reports were administered throughout the study).

### Inclusion criteria

Subjects were included in the study if they: 1) were free of musculoskeletal problems and potentially orthopaedic/neuromuscular limitations; 2) had a resting blood pressure below 140/90 mm Hg (subjects on antihypertensive medications (n = 6) maintained their medication throughout the study); 3) had no signs of cardiovascular/respiratory complications (at rest and during testing); 4) reported no tobacco use in the 6 months before the study or during the study; 5) did not assume aspirin, as cyclo-oxygenase can affect oxidant/antioxidant status, at least 1 week before exercise testing, and 6) were not consuming antioxidant compounds including vitamins, minerals, and medications (i.e., probucol, nebivolol, and anti-inflammatory agents).

### Exercise testing procedures

The following exercises were performed on a motorized treadmill (Laufergotest, Jaeger, Germany): a) An incremental exercise (IE) up to voluntary exhaustion (after 6 min warm-up at 10 km h^−1^ at 1% grade the speed of the belt was increased by 1 km h^−1^ every minute). Voluntary exhaustion was defined as maximal levels of self-perceived exertion using the validated Borg scale [39]. Peak oxygen uptake (V'O_2peak_) was determined as the average of the last 20 s values; b) Two 6-min constant-load exercises (CLE) of moderate (< gas exchange threshold, GET) and heavy (>GET) intensity respectively, separated by a 20-min recovery period. Pulmonary ventilation (V'E, expressed in BTPS - body temperature, pressure, and saturated), O_2_ uptake (V'O_2_), and CO_2_ output (V'CO_2_), both expressed in STPD (standard temperature, pressure, and dry), were determined breath-by-breath by a computerized metabolic cart (SensorMedics Vmax29c, Bilthoven, The Netherlands). Expiratory flow measurements were performed by a mass flow sensor (hot wire anemometer), calibrated before each experiment by a 3 litres syringe at three different flow rates. Tidal volume and V'E were calculated by integration of the flow tracings recorded at the mouth. V'O_2_ and V'CO_2_ were determined by continuously monitoring PO_2_ and PCO_2_ at the mouth throughout the respiratory cycle and from established mass balance equations, after alignment of the expiratory volume and expiratory gases tracings and A/D conversion. Calibration of O_2_ and CO_2_ analyzers was performed before each experiment by utilizing gas mixtures of known composition. Digital data were transmitted to a personal computer and stored on disk. Gas exchange ratio (R) was calculated as V'CO_2_/V'O_2_. Heart rate (HR) was determined by ECG. Blood pressure (BP) was measured using a standard cuff sphygmomanometer. Severe hypertension (systolic BP value >250 mmHg) or falling BP during exercise were considered criteria for the termination of the test.

### Blood sampling and analyses

Each subject reported to the laboratory at 9:00 a.m. after an overnight fast for blood sampling. Subjects abstained from alcohol and caffeine consumption for at least 24 h, and did not perform physical exercise for the 48 h before testing. Approximately 3 mL of blood was drawn from an antecubital vein, with subjects remaining supine. The blood samples were collected in heparinised Vacutainer® tubes, and plasma was separated by centrifuge (5702R, Eppendorf, Germany) at 1000 g for 10 min at 4°C. The plasma samples were then stored in multiple aliquots at −80°C until assayed. Samples were thawed only once before analyses, which were performed within two weeks from collection.

#### Thiobarbituric acid-reactive substances (TBARS)

A TBARS assay kit (Cayman Chemical, U.S.), which allows a rapid photometric detection of the thiobarbituric acid malondialdehyde (TBAMDA) adduct at 532 nm, was used. Samples were read by a microplate reader spectrophotometer (Infinite M200, Tecam, Austria). A linear calibration curve was computed from pure MDA-containing reactions.

#### Protein Carbonyls (PC)

Reactive species produced directly or indirectly through lipid peroxidation intermediates also may oxidatively modify proteins. The accumulation of oxidized proteins was measured by content of reactive carbonyls. A Protein Carbonyl assay kit (Cayman Chemical, U.S.) was used to evaluate colorimetrically-oxidized proteins. The samples were read at 370 nm, by a microplate reader spectrophotometer (Infinite M200, Tecam, Austria), as described in detail by the manufacturer. Oxidized proteins values obtained were normalized to the total protein concentration in the final pellet (absorbance reading at 280 nm), in order to consider protein loss during the washing steps, as suggested in the kit's user manual.

#### Total antioxidant capacity (TAC)

Plasma TAC was measured by an enzymatic assay kit (Cayman Chemical, U.S.) using a microplate reader spectrophotometer (Infinite M200, Tecam, Austria). This assay is based on the ability of antioxidants in the plasma to inhibit the oxidation of 2, 2′-azinobis (3-ethylbenzithiazoline) sulfonic acid (ABTS, Sigma) to the radical cation ABTS+ by a peroxidase. The amount of the produced ABTS+ has been assessed by measuring the absorbance signals at 705 nm. The antioxidants concentration is proportional to the suppression of the absorbance signal. TAC was evaluated by a trolox (6-hydroxy-2,5,7,8-tetramethylchroman-2-carboxylic acid, Aldrich) standard curve, and was expressed as trolox-equivalent antioxidant capacity concentration (mM).

### Urine sampling and analysis

Each subject reported to the laboratory at 9:00 a.m. after an overnight fast for urine sampling. All samples were collected by voluntary voiding in a sterile container provided to the subject. Aliquots of the urine were stored at −80°C until the analyses were performed.

#### 8-hydroxy-2-deoxy Guanosine (8-OH-dG)

8- hydroxy -2-deoxy guanosine (8-OH-dG) has been established as a marker of oxidative DNA damage. A commercially-available enzyme immunoassay EIA kit (Cayman Chemical, U.S.) was utilized. The EIA employs an anti-mouse IgG-coated plate and a tracer consisting of an 8-OH-dG-enzyme conjugate. This format has the advantage of providing low variability and increased sensitivity compared to assays that use antigen-coated plates. This assay is based on the competition between 8-hydroxy-2-deoxy guanosine and a 8-OH-dG acetylcholinesterase (AChE) conjugate (8-OH-dG Tracer) for a limited amount of 8-OH-dG. Because the concentration of the 8-OH-dG Tracer is held constant while the sample concentration of 8-OH-dG varies, the amount of 8-OH-dG Tracer that is able to bind to the 8-OH-dG monoclonal antibody will be inversely proportional to the concentration of 8-OH-dG in the sample. This antibody-8-OH-dG complex binds to goat polyclonal anti-mouse IgG that has been previously attached to the well. The plate is washed to remove any unbound reagents and then Ellman's Reagent (which contains the substrate to AChE) is added to the well. The product of this enzymatic reaction absorbs at 412 nm. The sample 8-OH-dG concentration is determined using a 8-OH-dG standard curve. Urinary concentrations of 8-OH-dG, as any urinary marker, vary considerably, therefore the urinary parameters are usually standardized based on the amount of creatinine excreted in the urine when the collection of the 24 h urine is not possible.

#### Creatinine

In the absence of renal disease, the excretion rate of creatinine in an individual is relatively constant. Thus, urinary creatinine levels may be used as an index of standardization for 8-OH-dG. A creatinine assay kit (Cayman Chemical, U.S.) was used to measure creatinine levels in urine samples. Samples were read by a microplate reader spectrophotometer (Infinite M200, Tecam, Austria). Creatinine concentration was determined using a creatinine standard curve.

### Training intervention

On the basis of the individual values of GET (expressed as % of the V'O_2peak_) obtained at PRE, participants were matched and assigned to either moderate-intensity continuous training group (MOD, n = 10) or high-intensity discontinuous training group (HIDT, n = 10). Each group undertook 8 weeks of training, three times a week. Three different types of training sessions were scheduled, with the total distance covered in each session being matched between the groups, in order to control for the training volume performed. For MOD, the sessions were as follows: a) 64.5 min at 70% GET, b) 58.5 min at 80% GET, and c) 54 min at 90% GET. For HIDT, the work-matched sessions were: a) 18×(1 min at 120% GET, 2 min at 65%), b) 18× (1 min at 130% GET, 2 min at 65%), and c) 18× (1 min at 140% GET, 2 min at 65%). In week 1 and 4, the participants performed only the session type “a” and “b”, while in week 8, the volume of session type “c” was reduced by decreasing the exercise duration (for MOD, 27 min at 90% GET; for HIDT, 9×1 min at 140% GET, 2 min at 65%).

### Statistical analysis

Data are expressed as Mean ± Standard Deviation. All results were tested for normal distribution using a Shapiro-Wilk test, and when the assumption of normality was not met, a natural log transformation was applied to reduce the bias due to non-uniformity of the error. Data from the resting oxidative stress measurements were analysed using a Two-Way ANOVA with repeated measures (group x training). Data from the oxidative stress kinetics were analysed using a Three-Way ANOVA with repeated measures (group x training x time). When statistical significance (p<0.05) was obtained for a main factor, a Bonferroni *post hoc* test was performed. The test-retest variability of the oxidative stress measures was analysed on the resting data in PRE and POST. In our hands the inter- and intra-assay coefficients of variation of the above-mentioned analyses were as follows: TBARS, 5.4% and 7.6%; PC, 4.8% and 11.8%; TAC, 8.5% and 7.7%, respectively.

## Results

### Resting values

**Figure 1:**
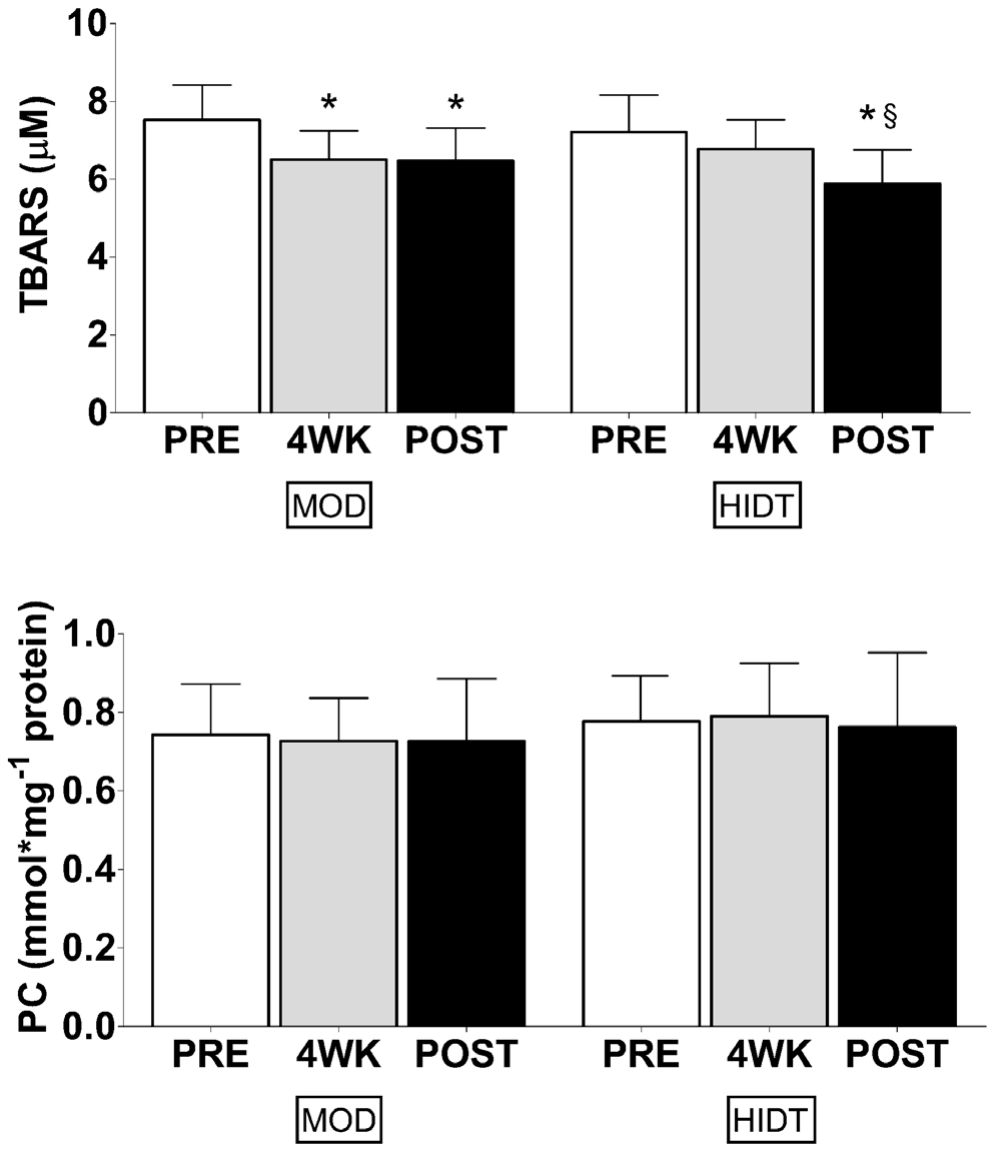
Effect of continuous moderate-intensity training (MOD) and high-intensity discontinuous training (HIDT) on thiobarbituric acid-reactive substances (TBARS) and protein carbonyls (PC). White bars represent pre-training (PRE) values, grey bars 4 weeks (4W) of training values and black bars post-training (POST) values. Values are expressed as means ± SD. * Significantly different from PRE (*P*<0.05). § Significantly different from 4WK (P<0.05).

**Figure 2:**
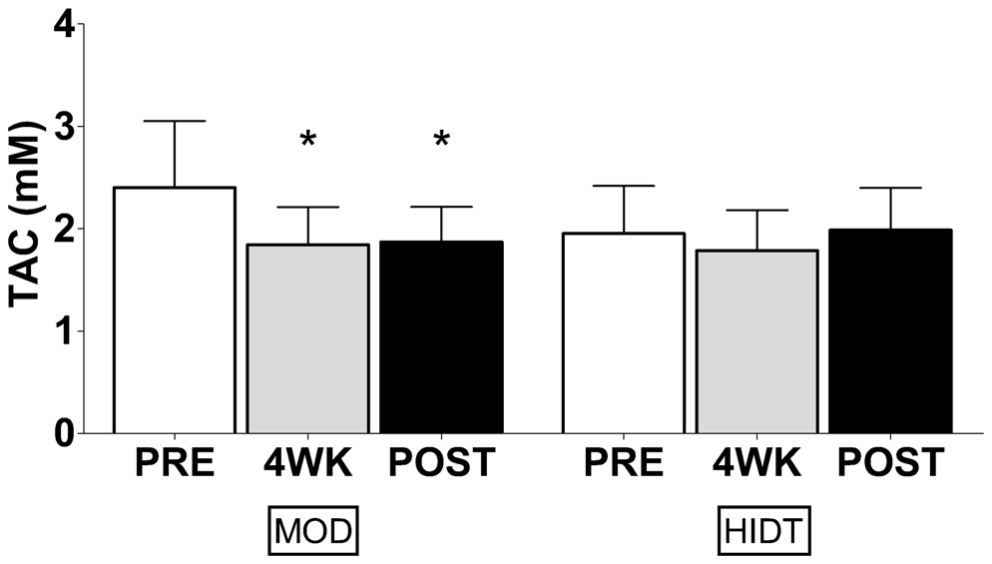
Effect of continuous moderate-intensity training (MOD) and high-intensity discontinuous training (HIDT) on total antioxidant capacity (TAC). White bars represent pre-training (PRE) values, grey bars 4 weeks (4W) of training values and black bars post-training (POST) values. Values are expressed as means ± SD. * Significantly different from PRE (P<0.05).

**Figure 3:**
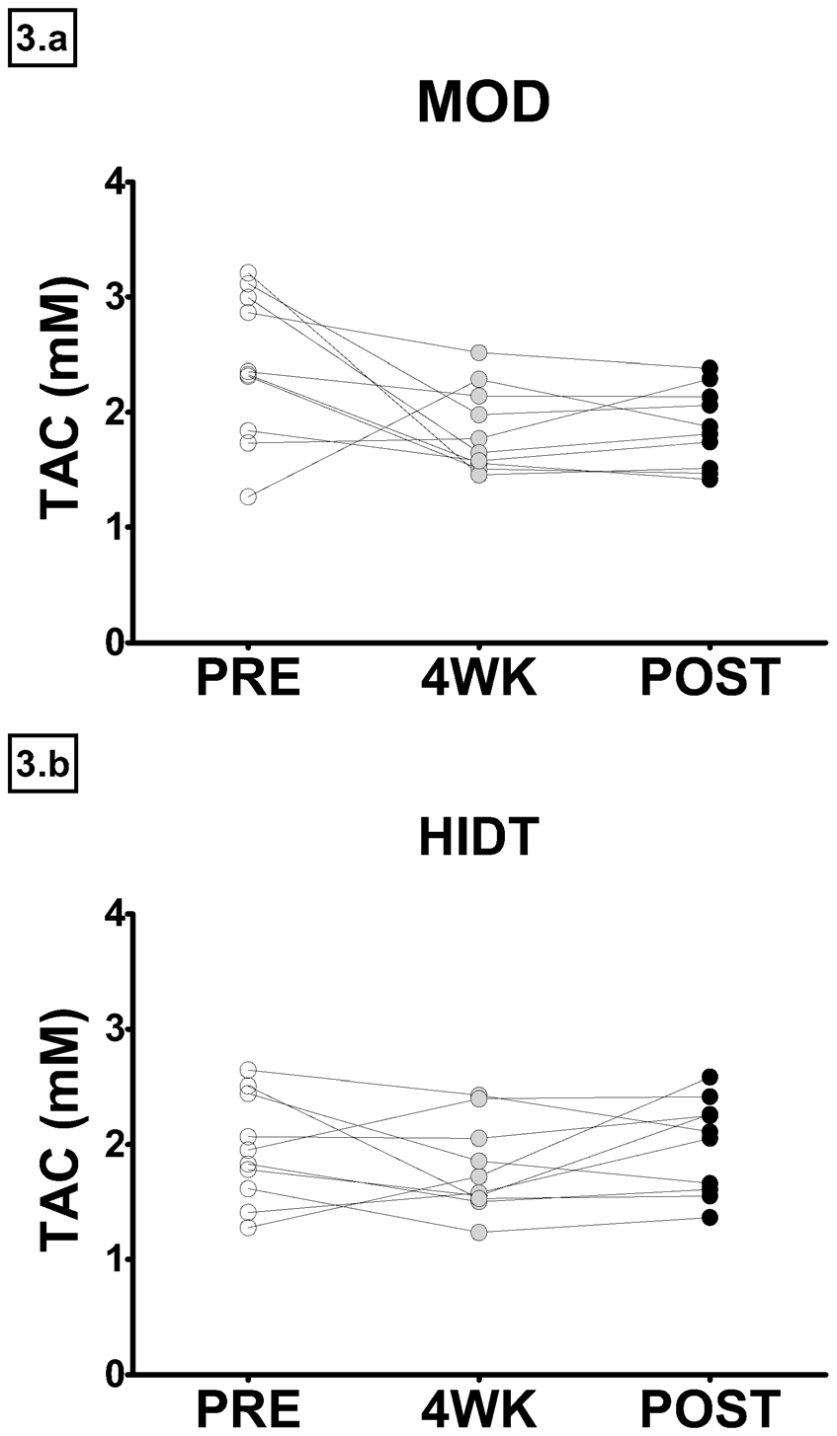
Individual changes in TAC value in MOD and HIDT. White squares represent pre-training (PRE) values and black squares represent post-training (POST) values.

**Figure 4:**
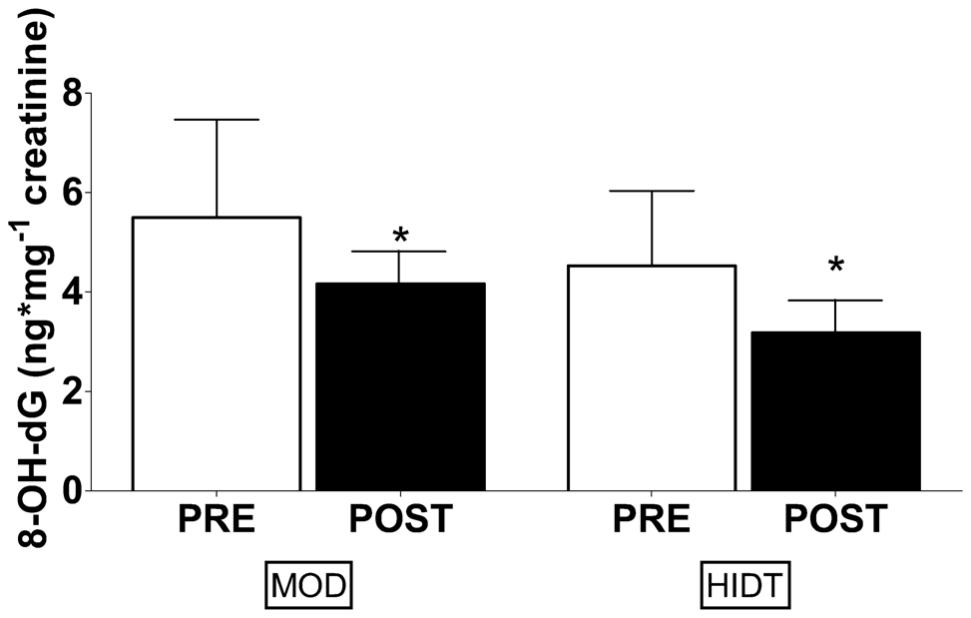
Effect of continuous moderate-intensity training (MOD) and high-intensity discontinuous training (HIDT) on oxidative damage of DNA measured by 8- hydroxy -2-deoxy guanosine (8-OH-dG). White bars represent pre-training (PRE) values and black bars are post-training (POST) values. Values are expressed as means ± SD. * Significantly different from PRE (P<0.05).

The resting plasma TBARS and PC concentrations are shown in Fig. 1. The upper panels show TBARS values before (PRE), after four weeks (4WK) and at the end (POST) of training in both MOD and HIDT group. In MOD, TBARS concentration declined significantly from PRE (7.53±0.30 µM) to 4WK (6.50±0.25 µM) and remained low in POST (6.46±0.27 µM). Also in HIDT, TBARS concentration declined from PRE (7.21±0.32 µM) to 4WK (6.78±0.25 µM), reaching a statistical significance at POST (5.85±0.46 µM). No significant differences were observed in TBARS concentration between MOD and HIDT in all conditions. The lower panels show PC values in PRE, 4WK and POST for both MOD and HIDT. Training did not significantly modify the PC concentration both in MOD (0.74±0.04, 0.73±0.04 and 0.73±0.05 nmol mg^−1^ protein in PRE, 4WK and POST respectively) and HIDT (0.78±0.08, 0.78±0.04 and 0.76±0.06 nmol mg^−1^ in PRE, 4WK and POST respectively). No significant differences were observed in the resting concentrations of PC between MOD e HIDT. In Fig. 2, resting plasma TAC values are shown. In MOD, TAC values resulted significantly reduced in 4WK (1.84±0.12 mM) respect to PRE (2.40±0.20 mM), without any other significant change in the last four weeks of training (1.87±0.11 mM, POST). In HIDT, TAC values were unaffected by training (1.95±0.15, 1.79±0.12 and 1.98±0.13 mM in PRE, 4WK and POST, respectively). No significant differences were observed in TAC between MOD e HIDT. In fig. 3 individual TAC values are shown. A large individual difference in resting TAC values among the subjects was observed at PRE both for MOD and HIDT. At POST, TAC values distribution was less scattered both for MOD and HIDT. The urinary levels of 8-OH-dG, biomarker of in vivo oxidative DNA base modifications, are shown in Fig. 4. The 8-OH-dG concentration significantly decreased from PRE (5.50±0.66 and 4.52±0.50 ng mg^−1^ creatinine in both MOD and HIDT, respectively) to POST (4.16±0.40 and 3.18±0.34 ng mg^−1^ creatinine in both MOD and HIDT, respectively). No significant differences in 8-OH-dG concentration were observed between HIDT and MOD.

### Kinetics of adjustment

**Figure 5:**
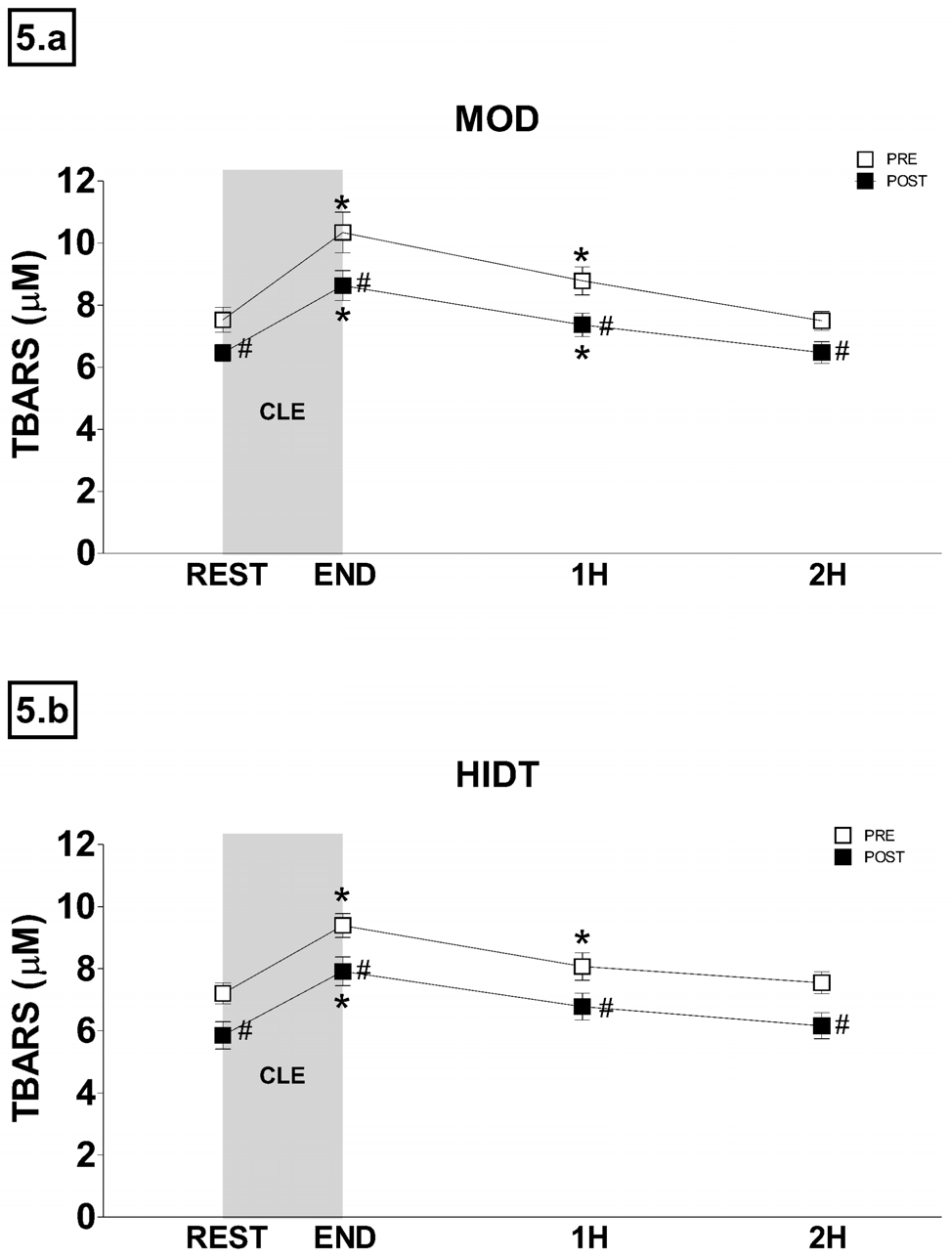
Time course changes of TBARS concentration recorded before (REST) and after (END, 1H and 2H) constant-load submaximal exercise trials (CLE). White squares indicate pre training (PRE) values and black squares post-training (POST) values. Values are expressed as means ± SD. *P<0.05 compared to REST. #P<0.05 compared to PRE.

The time course of TBARS and PC concentration changes obtained before, immediately after and at 1 and 2 hours of recovery from CLE carried out PRE and POST are shown in Fig. 5. In both groups and in all conditions TBARS concentration significantly increased immediately after exercise and returned toward resting levels thereafter. In MOD (Fig. 5a), as for PRE, TBARS concentration increased significantly in END (9.90±0.68 µM) and returned toward resting levels thereafter (8.28±0.57 µM and 7.62±0.63 µM in 1H and 2H respectively). As for POST, time course of TBARS concentration was similar but TBARS values were always significantly lower than PRE. In HIDT (Fig. 5b), the time course changes of TBARS were similar to those described for MOD. No significant differences were observed between MOD and HIDT.

**Figure 6:**
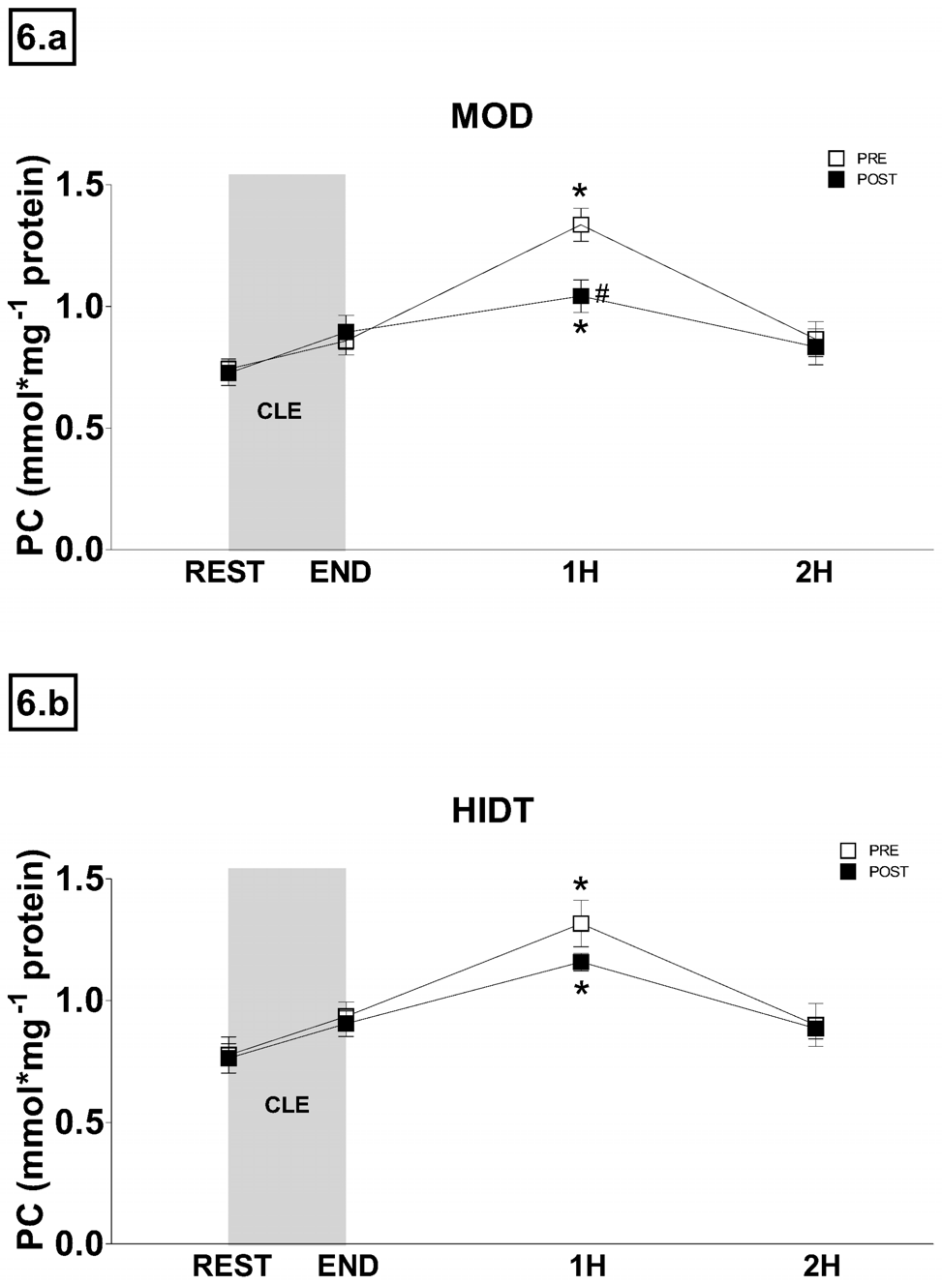
Time course changes of PC concentration recorded before (REST) and after (END, 1H and 2H) constant-load submaximal exercise trials (CLE). White squares indicate pre training (PRE) values and black squares post-training (POST) values. Values are expressed as means ± SD. *P<0.05 compared to basal value. #P<0.05 compared to PRE.

The time course changes of PC concentration are shown in Fig. 6. In PRE, PC increased progressively after CLE, reaching the significantly highest value at 1H (1.33±0.22 and 1.32±0.30 nmol mg^−1^ protein in both MOD and HIDT, respectively), and returning to resting values at 2H (0.87±0.07 and 0.90±0.09 nmol mg^−1^ protein in both MOD and HIDT, respectively). In POST, the time course of PC concentration was very similar but, as for MOD (Fig. 6a), the peak value reached at 1H (1.04±0.07 nmol mg^−1^ protein) was significantly lower than in PRE.

## Discussion

This study was designed to evaluate the oxidative stress response to high-intensity discontinuous training versus moderate-intensity continuous training in masters runners. The main findings are listed hereafter.

### TBARS resting values were significantly reduced after training both in MOD and HIDT

It is known that moderate intensity aerobic training such as those adopted by Fatouros et al. [28], i.e. 50–80% of HR_max_ for 16 weeks, or by Leeuwenburgh et al. [36] i.e. 75% of V'O_2max_ for 6 weeks, decreases resting lipid peroxidation levels. Our data are in agreement with these results since we observed in MOD a reduction of TBARS resting values. As for HIDT, the effects on lipid peroxidation levels are not well understood. A trend towards a reduction of resting plasma TBARS levels was shown in young subjects performing three sessions of HIDT within 1 week [40]. Our data confirm and extend these findings. We observed in masters runners a significant reduction in TBARS resting values only after 8 weeks of HIDT, but not after 4 weeks. Thus, the training duration seems to be an important variable affecting this adaptation. It is plausible that free radicals production and, consequently, lipid peroxidation induced by every single session of HIDT could be progressively reduced as observed within 1 week by Fisher et al. [40]. Moreover, it has been suggested that exercise training lowers resting lipid peroxidation by up-regulating antioxidant enzyme levels in tissues engaged in systematic exercise [41]. So, the 8 weeks of training could have allowed enough time for the antioxidant systems to reduce the acute damage of each single session of high-intensity training.

### No significant change in PC resting values was observed in both MOD and HIDT

It is know that aging is associated with increased free radical generation in the skeletal muscle and increased oxidative modification of protein, lipid, and DNA [32]. Moreover, some studies show that long-term training increases the macromolecular oxidative damage in elderly men. For example, Gonzalo-Calvo et al. [42] recently demonstrated that the level of carbonyl protein content in plasma and erythrocytes, are higher in a group of older men (>65 years) undergoing long-term training than in one group of sedentary subjects. Our data showed no significant changes in PC resting values after training in both MOD and HIDT, confirming previous reports on sedentary individuals undergoing 12 weeks of resistance training [43]. Now, oxidative modifications of protein (as accumulation of reactive carbonyl derivates) can serve as a tag to indicate which proteins need to be replaced [21]. Proteins are usually replaced by proteasome complex and an increased activity of proteasome could be an important factor that affects the rate of protein turnover and the remodeling of skeletal muscle [44]. Since it is known that exercise can induce the activity of proteasome complex and increase the rate of protein turnover, it is plausible that MOD and HIDT induced both the accumulation of reactive carbonyl derivates and the increase of damaged proteins proteolysis, leading to no significant changes in PC resting values. Therefore the unchanged PC resting values recorded in our study may be seen as a positive effect of both training protocols adopted.

### The accumulation of 8-OH-dG in urine was significantly reduced in MOD and HIDT

Several studies conducted submaximal aerobic exercise protocols under laboratory conditions to investigate DNA effects. DNA damage was neither seen after intense treadmill running in male subjects of different training status [45] nor in well-trained endurance athletes [46]. However, conflicting findings were reported when maximal exercise protocols, i.e. tests until exhaustion, were conducted under laboratory conditions. Increased levels of DNA strand breaks were observed after exhaustive treadmill running in subjects of different training status [26]. Moller et al. [47] demonstrated DNA strand breaks and oxidative DNA damage after an maximal cycle ergometer test under high altitude hypoxia, but not normal (normoxic) conditions. Furthermore, there were no differences in urinary 8-OHdG concentrations before and after supplementation with β-carotene within the 3 d following a cycle ergometer test to exhaustion [48].

As for training, a few studies have examined whether periods of intensified training affect genome stability. Increased urinary 8-OHdG levels were observed in 23 healthy males in response to a vigorous physical training programme (about 10 h of exercise for 30 d) [49] and in male long-distance runners throughout a training period for 8 d compared to a sedentary period [50]. However, in a longitudinal study no differences in urinary excretion of 8-OHdG between a group of long-distance runners and a sedentary control group were observed [51]. Our data showed a decrease (∼25%) in urinary 8-OH-dG excretion in both MOD and HIDT groups. These results could be explained by less DNA damage but also by activation of DNA repair processes. In fact, the activities of DNA damage-repairing enzymes are up-regulated by training [52]. To our knowledge this is the first study to evaluate oxidative DNA damage in humans following high intensity training. Contrary to our hypothesis the disturbances of cellular homeostasis caused by repeated variations of intensity in HIDT did not determine DNA damage significantly different from MOD. Therefore the beneficial adaptation observed may be independent from the intensity of training.

### The defences against oxidative damage were lowered only in MOD, not in HIDT

Skeletal muscle is a remarkably adaptive tissue that is capable of changing its morphological, physiological, and biochemical properties in response to various perturbations. The adaptations are accomplished by various signal transduction pathways that relay external stimuli to changes in intracellular enzyme activity and/or gene expression. Exercise-induced oxidative stress serves as an important signal to stimulate muscle adaptation of antioxidant systems via activation of the redox-sensitive signalling pathways [53]. While an acute bout of muscular contraction is sufficient to activate these pathways, up-regulation of enzyme protein synthesis requires cumulative effects from repeated bouts of exercise, that is, exercise training.

The effect of chronic exercise on redox status and antioxidant defence is a much-debated question. Chronic exercise training has been suggested to induce an increase of the activity of the antioxidant defence systems by animals [54] and humans studies [55], [28]. However, other studies have shown no change in sedentary individuals [53]–[56,], or even a decrease in antioxidant capacity with training [6], [36], [57], [58]. Results of the present study showed a significant decrease of the resting TAC values in MOD but not in the HIDT group. Even though it cannot be excluded that the different intensity of the training programmes could be responsible for this finding, an alternative explanation could be proposed.

TAC value can be considered a reliable biomarker of antioxidant defence, although it should be interpreted with some caution. It is well known that oxidative stress biomarkers are influenced by sex, age, lifestyle (i.e. smoking), dietary intake, previous strenuous exercise and/or training status. To overcome this inconvenience a “theoretically” homogeneous experimental group (males, no smokers, masters athletes) was chosen in present study. Nevertheless, large individual differences in resting TAC values among the subjects were observed at PRE (Fig. 3), resulting in a higher starting antioxidant defence level in MOD than in HIDT. Therefore, we believe that the significant training-induced decrease of TAC value observed in MOD might be attributed to a higher baseline. If we compare the participants' individual data before, during and after training it is easy to notice that training has induced a converging of TAC values towards an optimal level, especially in MOD (Fig. 3). In fact, participants who were characterized by low pre-training TAC values showed an increase of these, while subjects with high pre-training values showed a decrease. It is becoming increasingly clear that reactive species act in a hormetic manner [59] since an optimal ROS level is beneficial for the cell survival, whereas too little or too much ROS result in impaired physiological function. Therefore, excessive attenuation of ROS production, caused by high total antioxidant capacity values, if on one hand reduces oxidative damage on the other might be considered detrimental for cellular functionality.

### Kinetics of adjustment of oxidative stress biomarkers to acute exercise

There is an abundance of literature indicating that exercise increases the production of reactive oxygen species to a point that can exceed antioxidant defenses and thus cause oxidative stress [2], [5], [6], [60]. Few studies, however, have investigated with an adequate sampling time, the kinetics of adjustment of oxidative stress biomarkers after exercise. Michailidis et al. [61] after a specific aerobic exercise protocol have observed the highest value of TBARS and PC at 1 h and 4 h after exercise, respectively. In the present study the highest value of TBARS and PC was measured immediately at the end and 1 h after exercise, respectively. This shorter-lived response of PC and TBARS could be attributed, at least in part, to the lower intensity and shorter duration of the exercise protocol used in our study. More generally, the findings of the present study provide further evidence to the notion that non-muscle-damaging exercise induces alterations in redox homeostasis that last only few hours post exercise [62].

Moreover, our study also evaluated the effect of training protocols to the exercise-induced oxidative damage kinetics. According to Nikolaidis et al. [62], it becomes clear that the resting levels of many redox biomarkers return limited information compared to the ones modified by an acute exercise session. In other words, it may be easier to find an existing effect of a redox agent on body fluids redox status after exercise than at rest, simply because the stimulus of exercise may extend the magnitude and the duration of change in redox homeostasis. Both MOD and HIDT did not affect the time-course of plasma oxidative stress biomarkers. However, TBARS values at any time resulted significantly lower after training and PC peak value decreased after both HIDT and MOD resulting statistically different only for the latter. The decrease in the peak PC value observed might be a consequence of the activation of mechanisms induced by training procedures that more efficiently remove the oxidatively modified proteins from circulation.

## Limitations of the Study

This manuscript attempts to evaluate the effects of high-intensity discontinuous training on oxidative damage. Many approaches allow evaluation and demonstration of the participation of ROS in biochemical events. Indeed, the literature is replete with descriptions of different methodologies and approaches for these purposes. The only technique for direct detection of radicals is electron spin resonance, which allows the detection of relatively stable radicals. The indirect detection of ROS intervention is based on the dosage of specific end products resulting from the interaction of the ROS with biological macromolecules, such as DNA, proteins and lipid. The appearance of these end products serves as proof of the prior existence of ROS that left their footprints in the cell. The authors are aware that neither thiobarbituric acid ractive substances nor protein carbonyls or 8-hydroxy-2deoxy guanosine represent specific biomarkers of lipid peroxidation, protein oxidation or DNA base modifications. Nevertheless, we believe that our array of biomarkers is well able to characterize the oxidative status during the post-exercise period (and clearly equals or exceeds that of many similar investigations see for example [3] and [20]). It is possible that oxidative stress may have occurred in tissues aside from blood, such as skeletal muscle, which may be the ideal tissue when studying exercise stress. Of course, biopsies are required for obtaining samples for analyses, which is likely the reason why so few human investigations include the analysis of oxidative stress biomarkers in skeletal muscle.

## Conclusion

In conclusion, high-intensity discontinuous and continuous moderate-intensity training induced similar beneficial effects in masters runners, reducing the resting levels of oxidative stress biomarkers in plasma and urine. In addition, we provide further evidence that aerobic exercise induces alterations in redox homeostasis that last only few hours post exercise and are attenuated by training.

Therefore our hypothesis that HIDT might cause a higher level of exercise-induced oxidative stress compared to a workload-matched, moderate-intensity continuous training appears to be incorrect. It is also important to underline that these training adaptive responses appear effective even in middle-aged subjects.
